# Susceptibility of cancer patients and caregivers to online cancer misinformation: a qualitative interview study

**DOI:** 10.1007/s00520-026-11236-2

**Published:** 2026-09-18

**Authors:** Hind Mohamed, Jon Salsberg, Turki Alanzi, Mudathir Mohamed, Dervla Kelly

**Affiliations:** 1 School of Medicine, University of Limerick, Limerick, Ireland; 2 Health Information Management and Technology Department, Imam Abdulrahman Bin Faisal University, Dammam, Saudi Arabia; 3 E1 Health Cluster, King Fahad Specialist Hospital, Damman, Saudi Arabia; 4 Health Research Institute, University of Limerick, Limerick, V94 T9PX Ireland

**Keywords:** Cancer patients, Caregivers, Online misinformation

## Abstract

**Purpose:**

Despite the benefits of the internet for cancer care, there is also a substantial risk of exposure to misinformation. Our paper aims to explore the susceptibility of cancer patients and caregivers to online cancer misinformation.

**Method:**

We conducted semi-structured onsite interviews with nineteen cancer patients and six caregivers. We used descriptive-interpretive thematic analysis following an open-ended approach to investigate beliefs affecting susceptibility to online misinformation, the impact of cancer misinformation, the mitigation strategies, and the barriers and facilitators to safeguard against online misinformation. The theory of planned behaviour and the health belief model informed our data generation and analysis.

**Results:**

Six major themes emerged from the analysis: navigation/evaluation beliefs, belief in peer experiences, impact of cancer misinformation, trustworthy healthcare communication, and barriers and facilitators to safeguard against online misinformation. Participants reported using superficial methods to search and evaluate online cancer information to reduce their cognitive efforts. Peer experiences were considered a trustworthy source of cancer information. Cancer misinformation impacted the psychological and physical health outcomes of patients. The participants used trustworthy healthcare communication strategies and/or information sources to mitigate the effect of online misinformation. However, fear of confronting the physicians and reluctance to interfere with the consultation process were the main identified barriers. The participants recommended providing guidance on searching and evaluating credible information to help safeguard against online misinformation.

**Conclusion:**

Directing patients to well-known online national and international cancer information repositories and introducing evaluation checklists by clinicians may help patients safely search and evaluate online information.

**Supplementary Information:**

The online version contains supplementary material available at https://doi.org/10.1007/s00520-026-11236-2.

## Introduction

The burden of cancer is recognised globally as significant, with an impact on families, the economy, and society [1]. Due to the epidemiological transition phenomena, the global cancer incidence is expected to grow to 27.5 million new cancer cases, and cancer is expected to continue rising to 16.3 million cancer deaths by 2040 [1].

The diagnosis of cancer is emotionally traumatic, and it consumes a lot of energy from the patient and their family to accept and adapt to the new reality. Patients and their family members often need information and advice to cope with the situation and related challenges. They are confronted with many uncertainties and must make lifestyle decisions or support patients [2–4].

An integrative review of supportive care needs of people affected by cancer stated that cancer patients and caregivers were not satisfied with the existing possibilities for acquiring information regarding cancer [5]. This finding fits with other international studies highlighting the unmet information needs of cancer patients [6, 7].

Previous research indicated that patients tend to use multiple sources to obtain health information, with healthcare professionals [8], peers [8], and the internet [9] being the preferred sources of information.

Online information can be particularly effective where there are barriers to the use of traditional health information, especially for stigmatised conditions such as cancer [10]. Digital health is now considered a complement to current cancer care to meet key unmet needs, such as ‘support to help cope with a diagnosis’ and ‘cancer education and information’ [11]. Indeed, one study reported ‘information and education’ as the most needed digital health function for cancer patients and caregivers [12].

Digital health literacy (DHL) is the ability to use information and communication technologies to find, evaluate, create, and communicate health information, requiring both cognitive and technical skills. Information search is a problem-solving task that involves complex cognitive processes, such as inferences and decision-making [13]. Complex and/or confusing web-based health information is especially difficult for cancer patients since their cognitive abilities decrease because of cancer and chemotherapy treatment [14]. Previous research reported that cancer patients and caregivers struggle to identify reliable information resources and can be misled or misinterpret information online [15, 16].

A recent systematic review paper (2023) [16] focusing on information-seeking behaviour among cancer patients reported that twenty quantitative and only four qualitative studies were conducted since 2002. Notably, none of the qualitative papers have applied a theory to understand cancer patients’ information-seeking behaviour.

To our knowledge, our paper is the first to explore the susceptibility of cancer patients and caregivers to online misinformation using the theory of planned behaviour (TPB) and the health belief model (HBM). The TPB is a psychological (social-cognitive) theory that links beliefs to behaviour. TPB has three core constructs: behavioural beliefs, normative beliefs, and control beliefs. These beliefs together shape the individuals’ behavioural intentions [17]. The HBM model is a psychological model used to predict health behaviour by focusing on individuals’ beliefs and perceptions about their health. It consists of six primary cognitive constructs that influence behaviour, namely, perceived susceptibility, perceived severity, perceived benefits, perceived barriers, self-efficacy, and cues to action [18].

A recent systematic review of social media health information quality found that cancer was one of the topics for which the most quality concerns were raised [19]. Another systematic review on the prevalence of misinformation reported moderate rates of misinformation for noncommunicable diseases, such as cancer [20].

Given substantial evidence for the importance of reliable information in helping cancer patients with decision-making and coping with their illness [21], understanding patients’ information-seeking and evaluating behaviour is essential. As such, there is a need to explore how and why cancer patients are susceptible to online misinformation, as well as the safeguarding mechanisms that may reduce the impact of online misinformation. In addition, understanding the facilitators and barriers to safeguard against online misinformation could help the future design and implementation of DHL interventions.

## Aim

Explore cancer patients’ and caregivers’ susceptibility to online misinformation using the TPB and the HBM.

## Objectives

1. Understand participants’ beliefs affecting susceptibility to online misinformation.
2. Understand the impact of online misinformation on patients’ health outcomes.
3. Explore the participants’ mitigation strategies to safeguard against online misinformation.
4. Explore the participants’ barriers and facilitators to safeguard against online misinformation.

## Methods

COREQ (COnsolidated criteria for REporting Qualitative research) Checklist was used [22]. It includes a checklist of items that should be included in reports of qualitative research (Appendices).

### Study design

Semi-structured onsite interviews were conducted with cancer patients attending oncology clinics for follow-up and their caregivers at King Fahad Specialist Hospital (KFSH) in Dammam, Saudi Arabia. The researchers selected the semi-structured interview format because it allows them to pre-formulate the questions and adds more later during the questioning process [23].

### Sampling and recruitment

Following the principles of purposeful sampling [24], participants were recruited face-to-face with the oversight of the oncology consultant on duty. Paper-based posters were also used to facilitate recruitment. The first twenty-four patients who met these criteria were asked to participate in our study. Five of the patients declined (three men), and two others (two women) were too ill to be interviewed on the day of the appointment. Six caregivers agreed to participate in the study. The total number of patients and caregivers interviewed was twenty-five. Caregivers were interviewed separately from patients to allow them to speak openly.

### Inclusion and exclusion criteria

Table 1 presents the inclusion and exclusion criteria.

**Table 1:** Inclusion and exclusion criteria

Inclusion criteria	Exclusion criteria
1. Stable patients with a personal history of cancer	1. Patients with no personal history of cancer. Patients with personal history of cancer who are not not considered in remission
2. Or family/friend caregivers involved in helping care for patients with cancer for two or more hours per week providing emotional or other support	2. Or caregivers with no significant involvement in helping care, as defined by less than two hours per week of emotional or other support
3. Must be 18 years and older, male and female, and able to understand and answer in Arabic	3. Unable to give informed consent, unable to understand and answer in Arabic
4. Must have used the internet to search for cancer information	4. Never used the internet to search for cancer information

### Interview question design

A panel of five stakeholders has decided on questions to include in the interviews. The panel included clinicians, oncology consultants, and academics with expertise in health literacy. Experts brainstorm ideas independently, rank the best questions in a group, and vote to select the final list. Questions for the semi-structured interviews were open-ended, focusing on the following areas: beliefs affecting susceptibility to online cancer information, impact of cancer misinformation on patients’ health outcomes, mitigation strategies, and barriers to safeguard against online misinformation. The questionnaire initially had six questions. An additional question was added after a small number of pilot interviews to understand the facilitators to safeguard against online misinformation. We developed the interview guide in English to facilitate discussions with English-speaking researchers. HM translated this interview guide into Arabic as we anticipated many participants would want to be interviewed in Arabic (Native language). Participants were provided with an Arabic version of the topic guide before consultation. Appendices include the English version of the interview guide. Sociodemographic data were collected via a brief pre-interview questionnaire. Table 2 presents the participants’ characteristics.

**Table 2:** Participants’ characteristics

Participant category	Number of participants total *n* = 25
Cancer patient	19
Cancer caregiver	6
Age group	
30–40	5
41–50	8
51–60	7
More than 60	5
Gender	
Male	10
Female	15
Interview duration (minutes)	
15–30	4
30–45	15
46–60	6

### Data collection

A neutral female PhD researcher (HM) with no prior interaction with participants carried out the interviews in the oncology department/KFSH. No one else was present besides the participants and the researcher. Twenty-five interviews were completed with cancer patients and caregivers following written and verbal informed consent. The onsite interviews were conducted in cycles between 15 August and 15 October 2024 at a time and location convenient to the participants. Interviews were audio recorded, transcribed, and coded using a thematic analysis approach within the framework method, a combined deductive-inductive approach to data analysis [25]. The Framework method deductively utilised a semi-structured interview format with predetermined questions, serving as an agreed analytic framework, while also allowing for inductive discovery of unexpected aspects of the participants’ experiences of the phenomena [25]. The audio recordings were transcribed using Microsoft Word. Most of the interviews lasted 30–45 min. Saturation was used as a guiding principle to assess the adequacy of the purposive sample [26]. We assessed code saturation to ensure it was sufficient to understand the issues identified. Then, we assessed meaning saturation to ensure that no further dimensions, nuances, nor insights into the issues could be found [26].

### Data analysis

Audio recordings of the interviews were made with the participants’ consent and transcribed verbatim in Arabic. The transcriptions (namely the quotes) were translated forward and backward into English by the principal investigator (HM) (Researcher translator). The principal investigator is a professional in reading, writing, and speaking both Arabic and English, as well as a health literacy expert researcher familiar with qualitative research and the research topic. A bilingual health literacy expert consultant reviewed the translation (MM). The transcriptions were analysed by iteratively reading transcripts. Thematic analysis was conducted on the Arabic and English versions of the transcript data to ensure no changes occurred by translating before analysis, rather than analysing the original data and then translating the results. Five stages of the framework process were applied to the qualitative data analysis, including familiarization, thematic framework identification, indexing, charting, mapping, and interpretation [25]. One researcher conducted coding using NVIVO 14 software; a second researcher from a different professional background reviewed the codes to ensure inter-coder reliability and heighten reflexivity [27].

We conducted the new member-checking approach to ensure participants’ expertise was fully utilised. A researcher presented a strong draft of the findings, including the anonymous quotations, to some participants for feedback. Participants could clarify their quotes to ensure their experiences were conveyed correctly; this approach also helped them integrate their stories with those of other participants [28].

To ensure awareness of reflexivity that one’s own beliefs can affect interpretation [27], three additional members of the research team—a cancer survivor and an oncology physician—reviewed and refined the themes and categories, seeking clarity on language and suggesting additional work. A narrative description was provided for each theme describing susceptibility to online misinformation.

Given the potential value of theory as a heuristic method to contribute to qualitative analysis processes [29], data generation and analysis were informed by the TPB [17] and the HBM [18]. Emergent themes and categories were subsequently mapped onto the TPB and the HBM constructs. The mapping process relied on iteratively moving between the emergent themes and categories and the model constructs to build knowledge about applying each construct to the project data [30].

### Ethical considerations

All the procedures performed in the study involved human subjects. Ethical approval was obtained before data collection from the KFSH research ethics committee (23 April 2024/EXT0429). Clinical trial number: not applicable. Full disclosure was made regarding the investigation’s objective and the legal rights of those participating. Each participant in the interview was given a pseudonym to preserve their confidentiality and maintain their anonymity. Every participant provided their informed consent before starting the interview. Participation in the study was entirely voluntary. Data will be kept on a password-protected Outlook OneDrive account of the principal investigator (HM) for two years and then destroyed.

## Results

We identified six themes and nine categories under the TPB and HBM constructs (Table 3). The TPB covered the following themes: navigation/evaluation beliefs (behavioural beliefs), belief in peer experiences (normative beliefs), and trustworthy healthcare communication (control beliefs). The HBM covered the following themes and categories: impact of cancer misinformation (perceived susceptibility), psychological and physical burden (perceived severity), barriers to safeguard against online misinformation (perceived barriers), facilitators to safeguard against online misinformation (perceived benefits), empowerment for cancer self-management (self-efficacy), and providing guidance towards searching and evaluating credible information (cues to action). We integrated the findings around the commonly identified themes and categories to generate the digital cancer information-seeking pathway (Supplementary Fig. 1).

**Table 3:** TPB and HBM constructs, themes, and categories

TPB and HBM constructs	Themes	Categories
1. Behavioural beliefs	1. Navigation/Evaluation beliefs	1.1 Using superficial methods to search and evaluate online information
2. Normative beliefs	2. Belief in peer experiences	2.1 Trust in peer experiences
3. Perceived susceptibility	3. Impact of cancer misinformation	3.1 Psychological burden
4. Perceived severity		3.2 Physical burden
5. Control beliefs	4. Trustworthy healthcare communication	4.1 Trust in oncology healthcare professionals
6. Perceived barriers	5. Barriers to safeguard against online misinformation	5.1 Fear of confronting and challenging the physicians
		5.2 Reluctance to interfere with the consultation process
7. Perceived benefits	6. Facilitators to safeguard against online misinformation	6.1 Empowerment for cancer self-management
8. Self-efficacy		6.2 Providing guidance for searching and evaluating credible online information
9. Cues to action		

## Navigation/evaluation beliefs

### Using superficial methods to search and evaluate online cancer information

Most of the participants described searching on the first Google webpage to reduce their mental efforts and time, representing cognitive offloading behaviour.

“I find it easy to open the links presented on the first Google webpage” Patient 6

“I usually open the links randomly on the first Google webpage. I don’t have time to search beyond that” Caregiver 3

Most cancer patients and caregivers were noted to use superficial strategies to evaluate online cancer information. Cancer patients spoke about using self-justification approaches, while caregivers relied on the design of the websites.

“So, I check the reliability of cancer information according to my experience and common sense; if the information is realistic, then I trust it” Patient 3

“Website design and visuals usually attracted me to read and trust the written information” Caregiver 4.

## Belief in peer experiences

### Trust in peer experiences

Most of the participants considered peer experiences as a trustworthy source of cancer information. Cancer patients often value the knowledge and experience of other cancer patients. They believed that no one could understand and explain to them better than their peers.

“I believe in blogs presenting peer experiences; you know, we are travelling in the same boat and going on the same journey” Patient 19

## Impact of cancer misinformation on patients’ health

Most of the participants claimed that cancer misinformation typically led to delayed medical care and the abandonment of evidence-based treatments for unproven alternatives leading to rapid disease progression. These false claims also caused intense emotional distress.

### Psychological burden

Most of the participants spoke about the psychological consequences of misinformation on patients’ health. Misinformation was reported to be harmful and to cause anxiety and overthinking.

“They come up with these sensationalist stories online without realising that these are things which can negatively affect people. I feel worried, then I started overthinking about the information I read” Patient 9.

### Physical burden

In addition, most of the participants spoke about the effect of misinformation on patients’ health. Taking traditional medications instead of chemotherapy treatment has led to late presentation and poor prognosis.

“I tried an online herbal medicine called Cherimoya for a long period. When I went to the doctor, it was a very disappointing moment; cancer had spread to the liver and lung” Patient 10.

## Trustworthy healthcare communication

### Trust in oncology healthcare professionals

Most of the participants explained their trust in oncology healthcare professionals. Belief in the maxim that “the doctor knows best” sometimes negates the perceived value of seeking information online. Faith was also linked with the view that medical knowledge is difficult for lay people to understand.

“I only have a certain amount of intellect and education, and of course, doctors know the best about medical knowledge” Caregiver 6

“To be honest, when they said to me it’s cancer, at first and last, I’ll put it in the doctors’ hands. There is nothing like an ignorant man trying to learn everything by himself” Patient 10.

## Barriers to safeguard against online misinformation

Most of the participants were concerned about discussing online information with their physicians. The reported barriers were fear of being perceived as confronting their physicians and reluctance to interfere with the consultation process.

### Fear of being perceived as challenging or confronting their physicians

This fear often stems from “Iatrophobia,” which is an extreme fear of doctors, or simply a desire to be seen as a “good” patient. This hesitation causes most of the participants to withhold questions relevant to online misinformation.

“Doctors are very busy during clinic time, and lots of patients are waiting to be seen. I don’t want to put more burden” Caregiver 3

### Reluctance to interfere with the consultation process

Reluctance to interrupt a medical consultation usually stems from a sense of deference or the feeling that “the doctor knows best.” Asking lots of questions was seen as stressful moments that could increase the doctor’s burden.

“Asking many questions during the consultation could be annoying and interrupt the doctors” Patient 15

## Facilitators to safeguard against online misinformation

### Empowerment for cancer self-management

Most of the participants described searching cancer information online before visiting doctors as a facilitator for patient-doctor interactions and therefore improving patients’ safety. Online searching supports patients’ self-care by boosting confidence in taking ownership of their health.

“I need to have some information first before persuading to go see a doctor, I feel confident to interact with a doctor and understand my medical condition” Patient 15

### Providing guidance towards searching and evaluating credible information

Most of the participants emphasised the role of doctors in helping them use online information safely. However, there were discrepancies in the opinions between patients and caregivers. Cancer patients preferred introduction of high-quality websites to search cancer information while caregivers recommended learning how to evaluate online information.

“I don’t know where to search. I would recommend doctors directing me to high-quality cancer websites” Patient 16

“Doctors could help teaching us the tips on how to check the reliability of online health information” Caregiver 5

## Discussion

### Summary of the main findings

Participants reported using superficial methods to search and evaluate online information. Peer experiences were considered a trustworthy source of health information. We also found evidence that cancer misinformation retrieved online negatively affected patients’ psychological and physical health in some cases. The participants used trustworthy healthcare communication strategies with doctors to mitigate the effect of online misinformation. However, barriers to using online information safely included the fear of being perceived as confrontational with physicians and the reluctance to interfere with the consultation process. The participants recommended providing guidance by clinicians on searching and evaluating credible information to safeguard against online misinformation.

### Navigation/evaluation beliefs

Cancer information seekers in our study were using superficial methods to search and evaluate online information to reduce their cognitive burden. This finding was established in previous studies [31, 32]. This strategy would help the participants to cope with the perceived costs of information overload and credibility evaluation. Increasing public awareness of critical analysis of online information is necessary. DHL training and fact-checking would be beneficial for the identification of trustworthy online information.

### Beliefs about peer experiences

Our study identified peer experiences as trustworthy information sources for cancer patients. This pattern of information-seeking was reported in previous studies [8, 33] and is known as fortuitous information seeking, which is defined as searching for cancer information from others diagnosed with cancer. People with cancer reported blogging about their lived experience with cancer as a positive way to address the reality of their mortality [34]. Infrequently, acquired online information can lead to conflict if the patient values it above that of the doctor. Some of the major platforms, especially Facebook and Twitter, have recently pursued the aim of managing fake news by modifying their algorithms to improve the quality of their content and counteracting bots that disseminate misinformation [35]. Social media platforms can effectively minimise the impact of the automated dissemination of content using bots.

### The impact of online misinformation on patients’ health

Our study identified the psychological and physical impact of online misinformation on cancer patients. This finding was seen in previous studies [36, 37]. Lack of regulations on posting medical information on the internet poses inherent risks. Another risk is the commercially driven resources that could lead to false empowerment of patients towards self-diagnosis and treatment [36]. Strengthening healthcare regulations on posting commercially driven content by influencers and media organizations could help combat misinformation.

### Mitigation strategies to safeguard against online misinformation

Our findings indicated that physicians were the preferred source for checking the reliability of online information. This finding agrees with previous studies [38–40]. Clinicians can address cancer misconceptions by asking where patients found their information, then collaboratively reviewing the material to identify inaccuracies and clarify misunderstandings.

### Barriers and facilitators to safeguard against online misinformation

Fear of being perceived challenging the physicians, along with reluctance to interfere with the consultation process, were common barriers identified in our paper. This finding agrees with previous studies [41, 42]. Doctors often work under rigid time constraints, which prevent them from having in-depth conversations with their patients. An important reported facilitator to safeguard against online misinformation is providing guidance on how to search and evaluate credible online information. This approach was seen in a prior cancer e-health literacy intervention [43] and reported in a systematic review study as an effective DHL educational strategy [44]. Directing patients to well-known international/national online repositories of cancer information and introducing the Currency, Relevance, Authority, Accuracy, Purpose (CRAAP) framework by clinicians can help patients search and evaluate online information safely [45].

### Practice implications

Clinicians could adopt a range of strategies to help patients navigate online information safely. Simple strategies involve recommending trustworthy cancer websites during consultations and displaying short evaluation checklists. However, creating pre- and post-visit communications can also be helpful to address the pre-identified barriers. A trained nurse can triage online search questions and refer complex queries to clinicians and introduce a brief internet-use questionnaire during patient intake. This practice can help clinicians prioritize their time with high-risk groups. Integrating these strategies into routine clinical practice could bridge cancer patients’ DHL gap while reducing healthcare professionals’ burden during consultation sessions.

### Limitations

There are several limitations inherent in our paper. The study focuses on patients from a single hospital, limiting generalisability to broader populations. Future research can include a diverse sample across multiple healthcare settings. Using purposive sampling, where researchers choose participants with specific characteristics relevant to their study can intentionally create bias. However, this bias is usually planned and purposeful**;** as such, the study findings may not be generalisable. The study participants were all deliberate internet users, selected to understand their experiences of navigational challenges online. Addressing non-internet users’ needs considering the implementation of DHL interventions was not considered in our paper. Only six caregivers were included in our study. Future research could include more caregivers and compare their experiences with cancer patients.

## Conclusion

Most of the participants used superficial methods to search for and evaluate online information, which patients described as self-justification strategies and which caregivers attributed to website design. Most of the participants preferred trustworthy healthcare communication strategies to mitigate the effect of online misinformation. Directing patients to well-known online cancer information repositories and introducing evaluation checklists for caregivers by clinicians may help patients safely search and evaluate online information.

## Supplementary Information

Below is the link to the electronic supplementary material.

ESM 1 (PDF 473 KB)

ESM 2 (DOCX 54.8 KB)

## Data Availability

All relevant data are within the manuscript and its supporting information files.

## Abbreviations

DHL: Digital health literacy
KFSH: King Fahad specialist hospital
TPB: Theory of planned behaviour
HBM: Health belief model
