# Supplementary material for: Susceptibility of cancer patients and caregivers to online cancer misinformation: a qualitative interview study
Source: Support Care Cancer. 2026 Sep 18;34(10):987. doi: 10.1007/s00520-026-11236-2 (PMC13588904; doi:10.1007/s00520-026-11236-2)
Supplement: Supplementary file 2 [file 520_2026_11236_MOESM2_ESM.docx]

**Article title: Susceptibility of cancer patients and caregivers to online cancer misinformation: A qualitative interview study**

**Journal name: Supportive Care in Cancer**

**Author names**

Hind Mohamed^1^**,** Jon Salsberg ^1^, Turki Alanzi^2^, Mudathir Mohamed^3^, Dervla Kelly ^1,4^

**Affiliations**

1. School of Medicine, University of Limerick, Limerick, Ireland.

2. Health information management and technology department, Imam Abdulrahman Bin Faisal University, Dammam, Saudi Arabia

3. E1 Health cluster, King Fahad Specialist Hospital, Damman, Saudi Arabia.

4. ULCaN and HIST research clusters, Health Research Institute, University of limerick, Limerick, Ireland, V94 T9PX.

**Corresponding author email addresses**

hind-awdw@hotmail.com.

[20138148@studentmail.ul.ie](mailto:20138148@studentmail.ul.ie)

**Interview guide: Interview meeting topic guide for patients/caregivers**

**The following are the main areas/questions that have been asked in the interview meeting:**

1. How do you search for cancer information on the internet?

2. How do you evaluate the reliability of online cancer information?

3. What would you consider trustworthy source of online health information?

4. What issues did you suffer when exposed to misleading online information?

5. How do you check for the reliability of online cancer information?

6. What are the barriers you experienced when checking the reliability of online information?

7. What are the facilitators for checking the reliability of online information?

Impact of cancer misinformation

**Behavioural beliefs**

Using superficial methods to search and evaluate online information

**Perceived susceptibility**

**Navigation/evaluation beliefs**

Psychological and physical burden

Trust in peer experiences

**Perceived severity**

**Normative beliefs**

**Belief in peer experiences**

**Control beliefs**

Trust in oncology healthcare professionals

**Trustworthy Healthcare communication**

- Empowerment for cancer self-management

- Providing guidance towards searching credible information

-Introduction of technology-driven solutions

- Fear of being perceived as confronting their physicians

- Reluctance to interfere with the consultation process.

**Perceived benefits**

**Perceived barriers**

**Barriers to safeguard against online** **misinformation** misinformation

**Facilitators to safeguard against online misinformation**

**Self-efficacy**

**Cues to action**

**Fig1: Digital cancer information-seeking pathway**
